# Intra-Pancreatic Fat Deposition Versus Hepatic Fat in Relation to Incident Heart Failure: A Prospective Cohort Analysis

**DOI:** 10.31083/RCM52798

**Published:** 2026-09-16

**Authors:** Lishan Li, Zenghui Zhang, Siqi Gao, Bin Li, Shaojie Han, Can Lu, Zhaoyu Liu, Shaorong Wu, Jiangtao Yu, Jun Guo

**Affiliations:** ^1^Department of Cardiology, The First Affiliated Hospital, Jinan University, 510630 Guangzhou, Guangdong, China; ^2^The First Clinical Medical College, Jinan University, 510632 Guangzhou, Guangdong, China; ^3^State Key Laboratory of Bioactive Molecules and Druggability Assessment, Guangdong Basic Research Center of Excellence for Natural Bioactive Molecules and Discovery of Innovative Drugs, Department of Cardiology, The First Affiliated Hospital, Jinan University, 510630 Guangzhou, Guangdong, China; ^4^Medical Research Center, Guangdong Provincial Key Laboratory of Malignant Tumor Epigenetics and Gene Regulation, Sun Yat-Sen Memorial Hospital, Sun Yat-Sen University, 510120 Guangzhou, Guangdong, China; ^5^Clinic for General Internal Medicine and Cardiology, Catholic Medical Center Koblenz-Montabaur, 56068 Koblenz, Germany

**Keywords:** intra‐pancreatic fat deposition, hepatic fat, visceral fat, heart failure

## Abstract

**Background:**

Although liver fat has been extensively studied, its independent contribution to heart failure (HF) remains debated. Moreover, whether fat stored in different visceral organs confers comparable cardiovascular risk remains unclear. Therefore, this study aimed to examine the associations among intra-pancreatic fat deposition (IPFD), hepatic fat, and visceral adiposity and new-onset HF.

**Methods:**

This prospective analysis included 22,038 UK Biobank participants who were free of HF at baseline. Time-to-event associations of IPFD, liver fat, and total visceral fat volume with incident HF were estimated using Cox regression models.

**Results:**

Over a median follow-up of 13.6 years, 282 participants developed HF. Spline modeling suggested a monotonic increase in HF risk with higher IPFD, whereas liver fat showed a non-linear, inverted U-shaped pattern. In multivariable models adjusting for cardiometabolic risk factors, the highest IPFD tertile was associated with a greater risk of HF than the lowest tertile (hazard ratio [HR], 1.68; 95% confidence interval [CI], 1.15–2.46; *p* = 0.007), whereas hepatic fat was not. The association with IPFD remained significant after additional adjustment for pancreatic volume (HR, 1.62; 95% CI, 1.10–2.38), pancreatic iron (HR, 1.58; 95% CI, 1.07–2.33), HF polygenic risk score (HR, 1.58; 95% CI, 1.07–2.33), and the triglyceride–glucose (TyG) index (HR, 1.50; 95% CI, 1.00–2.26). Discrimination for incident HF was comparable between IPFD (area under the curve [AUC], 0.662; 95% CI, 0.635–0.689) and visceral fat volume (AUC, 0.663; 95% CI, 0.635–0.691).

**Conclusions:**

Greater pancreatic fat accumulation was independently associated with future HF, regardless of established cardiometabolic risk factors. These results suggest that IPFD may provide additional value for HF risk assessment and prevention-oriented metabolic profiling.

## 1. Introduction

The relationship between intra-pancreatic fat deposition (IPFD) and the onset of heart failure (HF) has yet to be clearly established. Obesity is a recognized determinant of HF and is included in established prediction instruments, such as the Weight, Age, Hypertension, Creatinine, HDL-C, Diabetes control, QRS duration, Myocardial infarction, and CABG (WATCH-DM) score and the Pooled Cohort equations to Prevent Heart Failure (PCP-HF) model [1,2,3,4]. However, excess adiposity is not a uniform exposure; individuals differ substantially in where fat is stored [5]. Visceral and subcutaneous adipose depots, for instance, have different relationships with cardiovascular outcomes [6]. Whether fat accumulation in particular visceral organs has organ-specific relevance for HF has not been fully clarified.

Magnetic resonance imaging proton-density fat fraction (MRI-PDFF) allows non-invasive quantification of ectopic lipid within visceral organs and provides a quantitative measure of organ steatosis [7]. Hepatic steatosis is closely related to obesity, metabolic syndrome, and cardiovascular morbidity [8]. Nonetheless, recent evidence indicates that liver disease activity may be more informative for major cardiovascular events than liver PDFF alone [9]. In comparison, the cardiovascular implications of IPFD remain poorly characterized. Because excess pancreatic fat is relatively common in the general population and is linked to metabolic syndrome [2,10], we used the UK Biobank cohort to compare pancreatic and hepatic fat deposition in relation to incident HF.

## 2. Materials and Methods

### 2.1 Study Design and Population

Data were sourced from the UK Biobank (https://www.ukbiobank.ac.uk/), an ongoing population-based cohort study launched in the United Kingdom in 2006, which currently includes more than 500,000 participants [11]. From 2006 to 2010, adults aged 40–70 years attended one of 22 assessment centers across Scotland, England, and Wales. Assessment at baseline included physical measurements, electronic questionnaires, nurse-led interviews, and biological sample collection. Additional sociodemographic, psychosocial, and health-related data were obtained through repeated assessment and linkage with national health records. All participants provided written informed consent.

Participants were not eligible for the present analysis if they had HF before baseline, lacked MRI-derived pancreas PDFF, liver PDFF, or visceral fat volume data (Fig. 1), or had missing information for prespecified covariates, including age, race, sex, education, blood pressure, smoking status, alcohol intake, hemoglobin A1c (HbA1c), C-reactive protein, estimated glomerular filtration rate (eGFR), total cholesterol (TC), low-density lipoprotein cholesterol (LDL-C), and coronary heart disease (CHD).

**Fig. 1.**
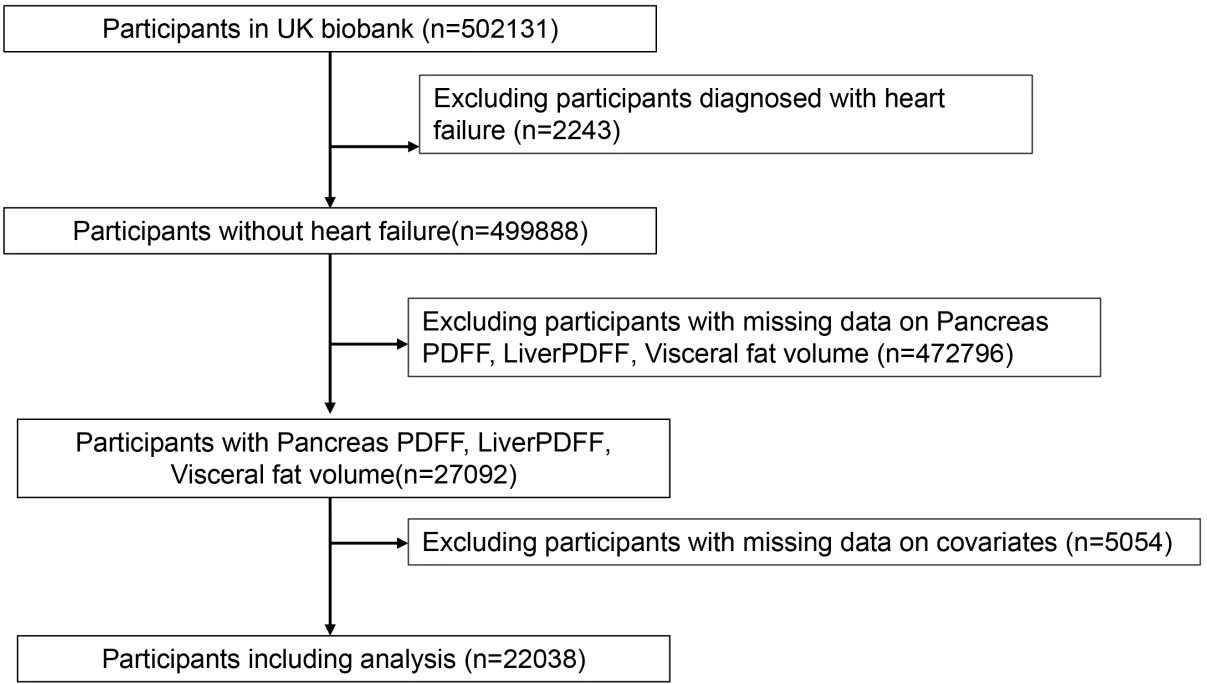
**Participant selection flowchart**. Note: PDFF, Proton Density Fat Fraction; Covariates included age, race, sex, education, current smoking, current drinking, diastolic blood pressure, systolic blood pressure, HbA1c, estimated glomerular filtration rate, C-reactive protein, TC, LDL-C, coronary heart disease. HbA1c, hemoglobin A1c; TC, total cholesterol; LDL-C, low-density lipoprotein cholesterol.

### 2.2 Assessment of Visceral Fat Deposition

Chemical-shift MRI provides a validated means of characterizing the amount and distribution of visceral adipose tissue [12,13]. By separating lipid and water signals, multi-echo Dixon imaging and magnetic resonance spectroscopy permit quantitative evaluation of tissue fat content without invasive procedures [14,15]. In this study, MRI-derived PDFF was treated as a continuous imaging phenotype. Visceral fat volume was obtained from high-resolution three-dimensional T1-weighted images acquired with a Siemens Aera 1.5-T scanner (Siemens Healthcare, Erlangen, Germany). Pancreatic and hepatic regions were segmented using a U-net algorithm trained on expert-labeled images, and organ-specific PDFF values were derived from single-slice multi-echo acquisitions. Further details regarding imaging protocols have been published previously [16,17].

### 2.3 Ascertainment of Genetic Risk

The HF polygenic risk score (PRS) was available from the UK Biobank and was constructed using 15 single-nucleotide polymorphisms (SNPs) previously associated with HF. Details of genotype imputation in the UK Biobank have been reported elsewhere [18].

This score was calculated as a weighted sum using the following formula:

PRS= (β__1_ × SNP__1_ + β__2_ × SNP__2_ + ⋯ + β__i_ × SNP__i_ + ⋯ + β__n_ × SNP__n_)

Here, n is the total SNP count, and β__i_ is the log odds ratio per allele for the association between SNP__i_ and HF.

### 2.4 Ascertainment of Heart Failure

New-onset HF was ascertained through self-reported diagnoses and hospital admission data linked to National Health Service records. HF was defined using International Classification of Diseases (ICD)-10 code I50 or UK Biobank self-report code 1076. Follow-up ended at the earliest occurrence of HF, death, or administrative censoring on October 31, 2022.

### 2.5 Assessment of Covariates

Baseline covariates were obtained from physical assessments, standardized questionnaires, and biochemical measurements. Demographic factors comprised age, sex, and race, with race classified as White or non-White. Lifestyle measures included educational attainment, smoking, and alcohol use. Clinical and laboratory variables included C-reactive protein, eGFR, TC, LDL-C, TyG index, blood pressure, and body mass index. eGFR was estimated using the 2021 Chronic Kidney Disease Epidemiology Collaboration creatinine equation, and the TyG index was computed as ln [fasting triglycerides (mg/dL) × plasma glucose (mg/dL) / 2] [19]. Comorbid conditions included CHD, chronic kidney disease, stroke, atrial fibrillation, valvular heart disease, and venous thromboembolism, as defined in **Supplementary Table 1**.

### 2.6 Statistical Analysis

For continuous variables, data are presented as means ± standard deviations. Categorical variables are reported as frequencies (percentages). Pancreas PDFF, liver PDFF, and visceral fat volume were grouped into tertiles for categorical analyses. To compare continuous variables between groups, we used analysis of variance or Wilcoxon rank-sum tests, as applicable. For categorical variables, we applied chi-square tests. Dose-response patterns between each continuous visceral fat measure and HF were explored using restricted cubic splines. Tertile-specific cumulative incidence curves were plotted and compared with log-rank tests. Cox proportional hazards regression was used to estimate HF risk associated with pancreas PDFF, liver PDFF, and visceral fat volume, both across tertiles and per standard deviation increment. Multivariable models included age, race, sex, education, current smoking, current alcohol use, blood pressure, HbA1c, C-reactive protein, eGFR, TC, LDL-C, and CHD. For pancreas PDFF, additional sequential models further included pancreatic volume, pancreatic iron, HF PRS, and TyG index. Receiver operating characteristic (ROC) analysis was applied to evaluate the ability of IPFD to discriminate incident HF. Robustness was examined by excluding participants followed for less than two years and by adding other HF-related conditions, including chronic kidney disease, stroke, atrial fibrillation, valvular heart disease, and venous thromboembolism. Analyses were performed in R statistical computing environment (Version 4.4.2, R Foundation for Statistical Computing, Vienna, Austria), and two-sided *p* values below 0.05 were considered statistically significant.

## 3. Results

### 3.1 Study Cohort

A total of 22,038 participants were included. Their mean age was 54.76 years, 97.10% were White and 51.50% were women. Across a median follow-up of 13.60 years, 282 participants (1.28%) experienced incident HF. Table 1 presents baseline characteristics by HF status. Individuals who developed HF tended to be more frequently male, were older, and were more likely to currently smoke than those who remained free of HF. They also had higher blood pressure, BMI, C-reactive protein, and HbA1c, more comorbid disease, and lower eGFR, LDL-C, TC, and HbA1c, more comorbid disease, and lower eGFR. Baseline profiles according to tertiles of pancreas PDFF, liver PDFF, and visceral fat volume are provided in **Supplementary Tables 2–4**. Because many characteristics varied across ectopic fat categories, these factors were considered in adjusted models. The highest tertile for each visceral fat measure corresponded to a higher incidence rate of HF (**Supplementary Fig. 1**).

**Table 1.** **Baseline characteristics of participants stratified by incident heart failure**.

Variables		All	Without incident HF	Incident HF	*p *value
n		22,038	21,756	282	
Age, years, mean (SD)		54.76 (7.45)	54.69 (7.44)	60.35 (6.28)	<0.001
Sex, n (%)					<0.001
	Female	11,354 (51.50)	11,288 (51.90)	66 (23.40)	
	Male	10,684 (48.50)	10,468 (48.10)	216 (76.60)	
White, n (%)		21,396 (97.10)	21,119 (97.07)	277 (98.23)	0.252
University education degree, n (%)		10,271 (46.60)	10,170 (46.70)	101 (35.80)	<0.001
Systolic blood pressure, mmHg, mean (SD)		136.75 (18.84)	136.65 (18.79)	144.56 (21.12)	<0.001
Diastolic blood pressure, mmHg, mean (SD)		81.42 (10.45)	81.39 (10.43)	83.59 (11.67)	<0.001
HbA1c, mmol/mol, Mean (SD)		34.97 (5.16)	34.95 (5.14)	36.83 (6.27)	<0.001
Smokers, n (%)		8541 (38.80)	8393 (38.60)	148 (52.50)	<0.001
Drinkers, n (%)		21,481 (97.50)	21,204 (97.50)	277 (98.20)	0.534
Coronary heart disease, n (%)		605 (2.70)	558 (2.60)	47 (16.70)	<0.001
eGFR, mL/min/1.73 m^2^, mean (SD)		91.95 (12.15)	92.01 (12.14)	87.40 (12.01)	<0.001
BMI, kg/m^2^, Mean (SD)		26.56 (4.15)	26.54 (4.14)	28.19 (4.36)	<0.001
C reactive protein, mg/L, mean (SD)		2.01 (3.37)	2.00 (3.32)	2.83 (6.00)	<0.001
Total Cholesterol, mmol/L, Mean (SD)		5.73 (1.08)	5.73 (1.08)	5.47 (1.11)	<0.001
Low-Density Lipoprotein Cholesterol, mmol/L, Mean (SD)		3.59 (0.83)	3.59 (0.83)	3.46 (0.83)	0.010
Chronic kidney disease, n (%)		78 (0.35)	75 (0.34)	3 (1.06)	0.130
Stroke, n (%)		141 (0.63)	137 (0.62)	4 (1.41)	0.202
Hypertension, n (%)		4342 (19.70)	4227 (19.42)	115 (40.78)	<0.001
Valvular heart disease, n (%)		154 (0.69)	148 (0.68)	6 (2.12)	0.011
Atrial fibrillation, n (%)		127 (0.57)	114 (0.52)	13 (4.60)	<0.001
Venous thromboembolism, n (%)		255 (1.15)	250 (1.14)	5 (1.77)	0.488

Note: Abbreviation definitions: eGFR, estimated glomerular filtration rate; BMI, body mass index.

### 3.2 Visceral Fat Deposition and Heart Failure Risk

Spline analyses indicated that HF risk increased with higher pancreas PDFF and visceral fat volume, while liver PDFF displayed an inverted U-shaped association (Fig. 2). Cumulative incidence plots showed increasing HF occurrence across tertiles of visceral fat measures, with the greatest event burden in the highest tertile (Fig. 3). In models adjusted for age, race, sex, education, blood pressure, smoking, alcohol use, HbA1c, C-reactive protein, eGFR, TC, LDL-C, and CHD, participants in the highest tertile of pancreas PDFF and visceral fat volume had elevated HF risk compared with those in the lowest tertile (pancreas PDFF: HR, 1.68; 95% CI, 1.15–2.46; *p* = 0.007; visceral fat volume: HR, 1.82; 95% CI, 1.19–2.78; *p* = 0.005) (Fig. 4A). Findings were consistent when these exposures were modeled per standard deviation increment (pancreas PDFF: HR, 1.02; 95% CI, 1.01–1.03; *p* < 0.001; visceral fat volume: HR, 1.08; 95% CI, 1.02–1.14; *p* = 0.007) (Fig. 4B). Liver PDFF, however, did not show an independent association after multivariable adjustment (tertile 3 vs. tertile 1: HR, 0.86; 95% CI, 0.63–1.18; *p* = 0.352; per SD: HR, 0.99; 95% CI, 0.97–1.02; *p* = 0.577) (Fig. 4A,B). The pancreas PDFF-HF association remained evident after sequential adjustment for pancreatic volume, pancreatic iron, HF PRS, and TyG index, but not after BMI adjustment (Fig. 5). ROC curves demonstrated similar HF discrimination for pancreas PDFF (AUC, 0.662; 95% CI, 0.635–0.689) and visceral fat volume (AUC, 0.663; 95% CI, 0.635–0.691) (Fig. 6).

**Fig. 2.**
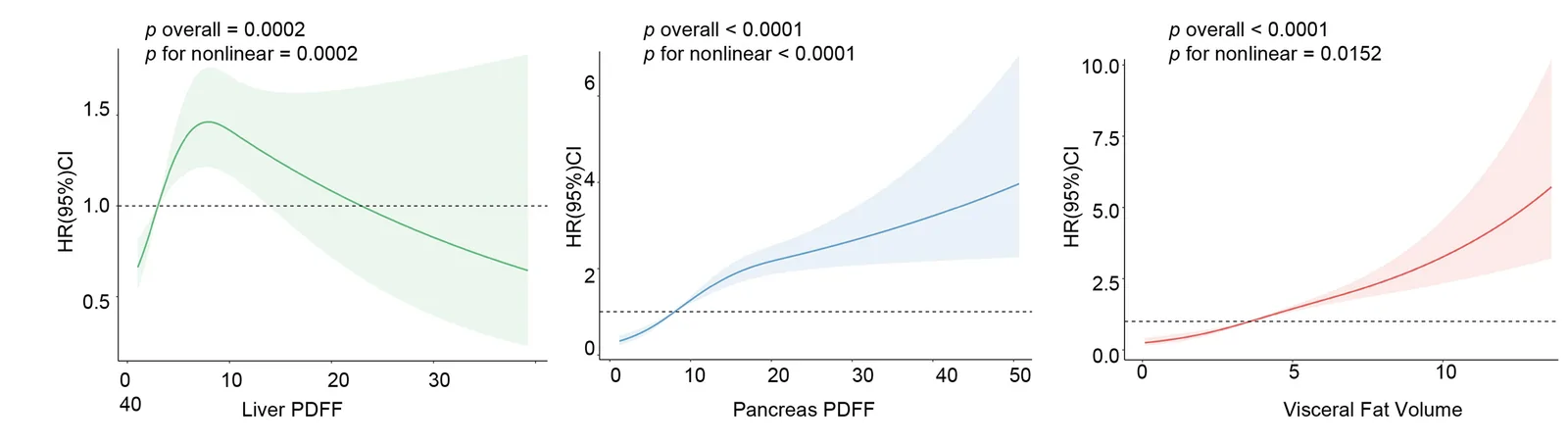
**Restricted cubic spline analysis of visceral fat measures and heart failure risk**. Note: PDFF, proton density fat fraction.

**Fig. 3.**
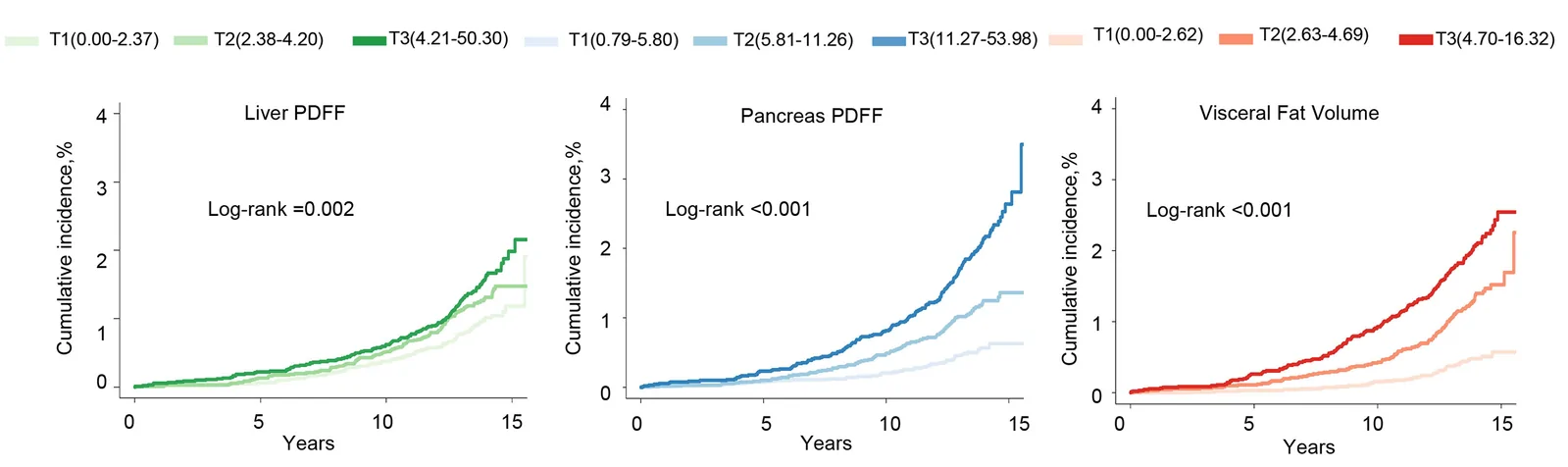
**Cumulative incidence of heart failure by tertiles of visceral fat measures**. Note: Cumulative incidence curves for the three tertile groups were compared using the log-rank test, with log-rank *p*-values shown.

**Fig. 4.**
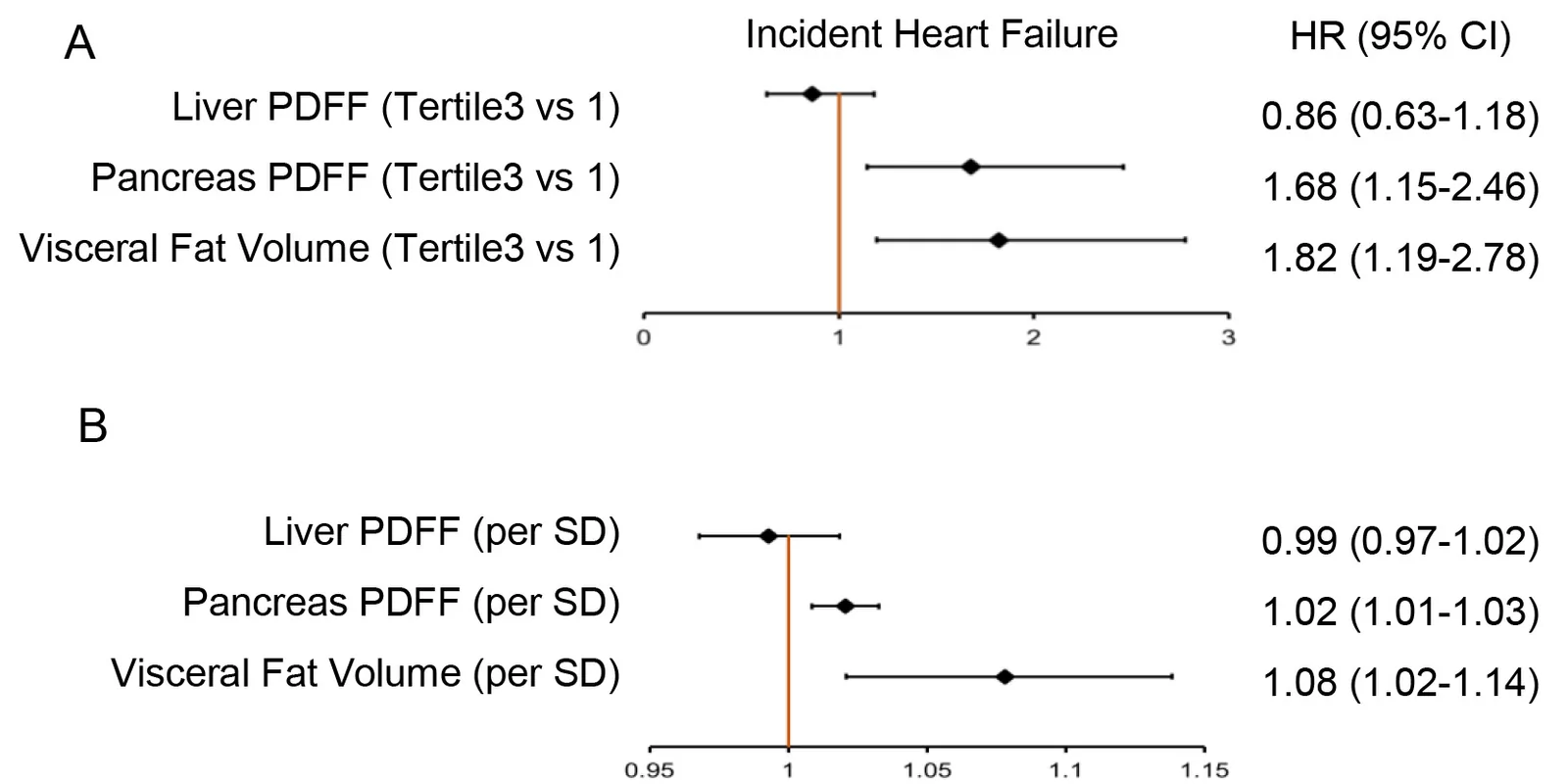
**Adjusted associations of visceral fat measures with incident heart failure**. Note: (A) Visceral fat tertiles: T1 (lowest). (B) Visceral fat as continuous variable, per SD. Models adjusted for age, race, sex, education, current smoking, current drinking, diastolic blood pressure, systolic blood pressure, HbA1c, estimated glomerular filtration rate, C-reactive protein, TC, LDL-C, coronary heart disease.

**Fig. 5.**
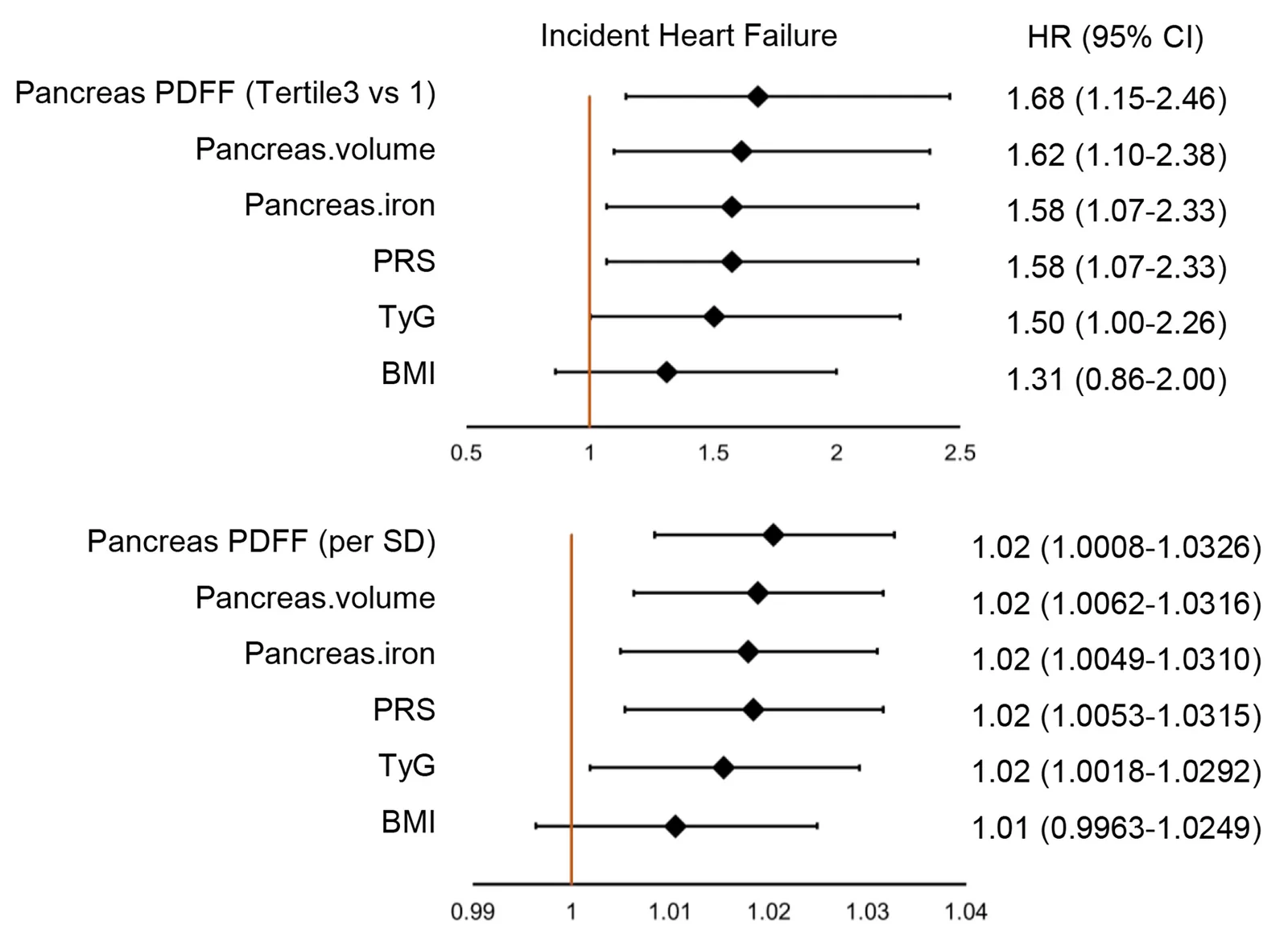
**Sequentially adjusted association between pancreatic fat and incident heart failure**. Note: (A) Pancreas fat categorized by tertile. (B) Pancreas fat analyzed continuously (per SD). Baseline models adjusted for age, race, sex, education, current smoking, current drinking, diastolic blood pressure, systolic blood pressure, HbA1c, estimated glomerular filtration rate, C-reactive protein, TC, LDL-C, coronary heart disease, with sequential, additional adjustments for pancreas volume, pancreas iron, heart failure polygenic risk score (PRS), triglyceride-glucose index (TyG), BMI.

**Fig. 6.**
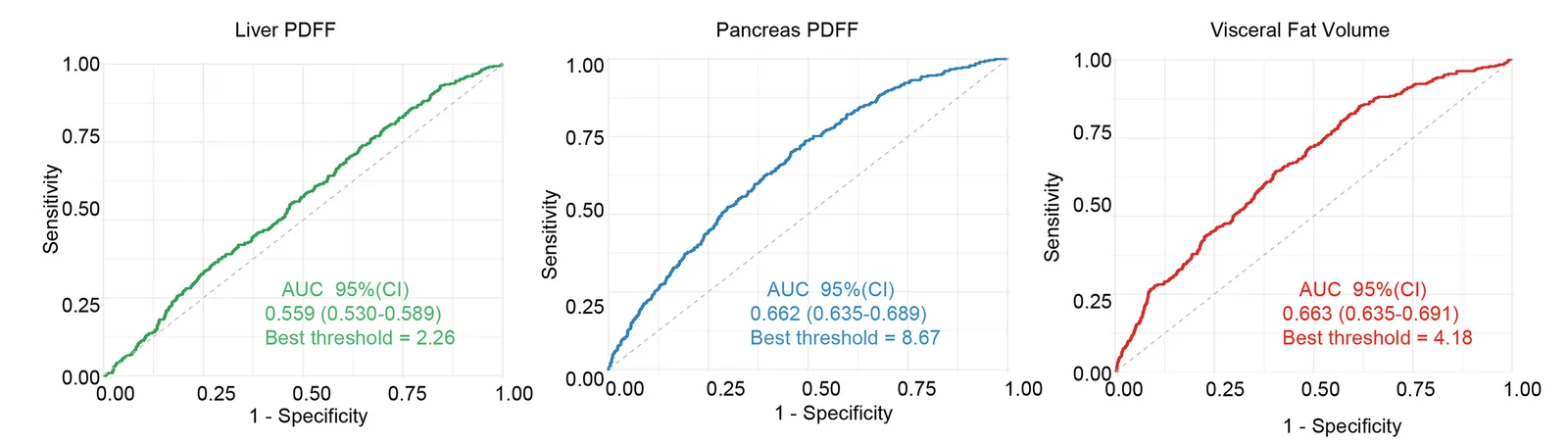
**ROC curves evaluating visceral fat measures for incident heart failure**. Note: ROC curves assessed the ability of liver PDFF, pancreas PDFF, and visceral fat volume to discriminate incident heart failure. The area under the curve (AUC) reflects the discriminative accuracy of each index. 95% confidence intervals for AUC estimates are shown to indicate the precision of these measures. ROC, receiver operating characteristic.

### 3.3 Sensitivity Analyses

Sensitivity analyses supported the robustness of the main results. When participants with follow-up shorter than two years were removed, the hazard estimates for the visceral fat measures changed only modestly (**Supplementary Table 5**). Results were also materially unchanged after adding further HF-related covariates, indicating that the primary findings were stable (**Supplementary Table 5**).

## 4. Discussion

Several findings emerged from this prospective cohort analysis. Higher pancreatic fat was related to greater subsequent HF risk, whereas the apparent relationship for hepatic fat weakened after accounting for conventional HF risk factors. The association between IPFD and HF persisted after considering pancreatic size, pancreatic iron, genetic predisposition, and insulin resistance. In addition, pancreatic PDFF showed HF discrimination similar to that of total visceral fat volume.

Ectopic lipid storage is biologically linked with metabolic syndrome and insulin resistance [20]. Accumulation of lipid intermediates, such as diacylglycerols and ceramides, can stimulate inflammatory and stress-related signaling pathways, including Toll-like receptor 4, protein kinase C, and JNK-1, thereby disrupting insulin signaling in metabolically active tissues. Pancreatic lipid accumulation may therefore contribute to lipotoxicity, impaired insulin action, and β-cell dysfunction, creating a metabolic milieu that favors HF development. Although homeostatic model assessment of insulin resistance (HOMA-IR) is widely used to estimate insulin resistance, the TyG index is easier to obtain in large clinical and epidemiological datasets and has been related to metabolic syndrome, stroke, and chronic coronary artery disease [21].

The cardiovascular consequences of obesity are well recognized [22], but attention has increasingly shifted toward the distribution and biological activity of adipose depots. Visceral adiposity may impose greater cardiometabolic burden than subcutaneous fat [23]. Although non-alcoholic fatty liver disease (NAFLD) has been repeatedly associated with cardiovascular risk [24,25], growing evidence suggests that fibrosis or active liver disease may be more relevant to cardiovascular progression than hepatic fat quantity alone [9]. Our findings are consistent with this concept and further suggest that pancreatic fat, given the organ’s endocrine and exocrine roles, may be closely tied to insulin resistance, systemic inflammation, and lipotoxic injury.

Compared to NAFLD, intra-pancreatic fat deposition and iron overload are emerging yet under-characterized clinical phenomena [26]. Intra-pancreatic fat deposition has been independently associated with both aortic intima-media thickening and increased epicardial adipose tissue [27]. Epicardial adipose tissue functions as a metabolically active fat depot that shares a microcirculatory milieu with the coronary arteries and myocardium [20], enabling local paracrine effects through the secretion of pro-inflammatory cytokines and bioactive molecules such as adiponectin, tumor necrosis factor-α (TNF-α), interleukin-1 (IL-1), interleukin-6 (IL-6), monocyte chemoattractant protein-1 (MCP-1), and nerve growth factor. Many of these inflammatory molecules are thought to originate from infiltrating macrophages. Locally produced cytokines diminish insulin-induced vasodilation and capillary recruitment, thereby promoting vasoconstriction. These factors may exert long-term effects on cardiovascular function and morphology, thus contributing to the pathogenesis of cardiovascular disease [28]. Pancreatic iron overload may also induce acinar cell death and exocrine dysfunction, processes associated with increased cardiovascular events [29]. In addition, type 2 diabetes is more than twice as common among individuals with non-alcoholic fatty pancreas disease [2]. Because type 2 diabetes involves both insulin resistance and β-cell dysfunction, the association between IPFD and β-cell impairment provides a plausible mechanistic pathway [30,31]. In the present study, IPFD remained positively associated with HF after adjustment for pancreatic iron overload and insulin resistance

Previous work has connected IPFD with markers of subclinical vascular injury, including greater carotid and aortic intima-media thickness and increased arterial stiffness [32]. Findings for clinically manifest cardiac disease, however, have been mixed. One 10-year cohort study published in 2022 did not identify a significant association between IPFD and incident ischemic heart disease, potentially because the number of events was small (20 of 631 participants, 3.2%) [33]. Likewise, a donor-based study of 55 individuals may have lacked sufficient power, despite observing a tendency toward more ischemic heart disease among those with histologically verified IPFD [34]. Another study among patients undergoing computed tomography (CT) angiography found no statistically significant relationship between IPFD and cardiovascular disease [35]. Conversely, Meloni et al. [36] observed higher IPFD among participants with cardiac complications, although that analysis was performed in a thalassemia major population and included only five HF cases. By leveraging a large and standardized imaging cohort, our study helps overcome some of these limitations and supports a positive association between IPFD and incident HF. These data indicate that pancreatic fat may help identify people at increased HF risk.

A further observation was that pancreatic PDFF discriminated HF to a degree comparable with total visceral fat volume. This finding supports the possibility that pancreatic fat is not merely a surrogate for overall visceral adiposity but may represent a metabolically informative depot relevant to cardiac dysfunction. Because the pancreas has a central role in glucose and energy homeostasis, IPFD may reflect both systemic energy excess and local metabolic stress related to inflammation and insulin resistance. In this context, IPFD may characterize the metabolic quality of visceral adiposity in addition to its quantity. Multiple pathways could plausibly connect pancreatic fat with cardiomyocyte dysfunction [37]. When fatty acid oxidation becomes insufficient, excess lipid may be diverted into non-oxidative pathways and accumulate in cardiomyocytes. Increased fatty acid exposure can heighten mitochondrial workload, while saturated non-esterified fatty acids such as palmitate may directly damage cardiac cells. Myocardial insulin resistance may further impair substrate utilization, promote toxic lipid deposition, worsen ischemia-related dysfunction, reduce mitochondrial efficiency, and increase reactive oxygen species, lipid peroxidation, and ferroptosis. Further mechanistic studies are needed to define how pancreatic fat affects cardiac structure and function.

## 5. Strengths and Limitations

This work has notable strengths. The use of MRI-based quantification allowed hepatic and pancreatic fat to be assessed as continuous imaging traits rather than relying on crude disease categories. In addition, the analysis was based on a large prospective UK Biobank dataset with linked outcome information. These features provided an opportunity to evaluate IPFD and incident HF with improved phenotypic precision and longitudinal follow-up.

Several limitations warrant mention. The cohort consisted mainly of middle-aged and older adults of European ancestry, so the findings may not directly generalize to populations with different ethnic backgrounds or age distributions. Pancreas PDFF, liver PDFF, and visceral fat volume were available only at baseline, which precluded evaluation of longitudinal changes in ectopic fat. Finally, despite adjustment for a broad set of covariates, the possibility of residual or unmeasured confounding remains.

## 6. Conclusions

In summary, pancreatic fat accumulation was associated with incident HF independently of traditional cardiometabolic risk factors. Measuring pancreas PDFF may enhance HF risk assessment, support identification of individuals who could benefit from targeted metabolic prevention, and complement conventional evaluation of visceral adiposity. Prospective interventional studies are needed to determine whether pancreas PDFF has clinical utility for guiding HF prevention strategies.

## Data Availability

The anonymized data analyzed in this work were accessed through official UK Biobank resources.
