# Supplementary material for: Intra-Pancreatic Fat Deposition Versus Hepatic Fat in Relation to Incident Heart Failure: A Prospective Cohort Analysis
Source: Rev Cardiovasc Med. 2026 Sep 16;27(9):52798. doi: 10.31083/RCM52798 (PMC13653158; doi:10.31083/RCM52798)
Supplement: Supplementary file 1 [file 2153-8174-27-9-52798-s1.zip › Supplementary Material.docx]

**Supplemental Material**

Intra-Pancreatic Fat Deposition Versus Hepatic Fat in Relation to Incident Heart Failure: A Prospective Cohort Analysis

Content of Supplemental Material

**Supplementary Table 1.** Definitions of heart failure and associated comorbidities.

**Supplementary Table 2.** Baseline characteristics of participants stratified by pancreas PDFF tertile.

**Supplementary Table 3.** Baseline characteristics of participants stratified by liver PDFF tertile.

**Supplementary Table 4.** Baseline characteristics of participants stratified by visceral fat volume tertile.

**Supplementary Table 5.** Sensitivity analysis of the associations between visceral fat and heart failure.

**Supplementary Fig. 1.** Association between tertiles of visceral fat and incident heart failure.

**Supplementary Table 1. Definitions of heart failure and associated comorbidities.**

| Diagnosis | ICD-10 codes | Self-reported diseases (Field ID 20002) |
| --- | --- | --- |
| Heart failure | I50 | 1076 |
| Venous Thromboembolism | I80.1, I80.2, I80.3, I80.0, I80.8, I80.9, I81, I82.0, I82.2, I82.3, I82.8, I82.9, I67.6, O22.5, O22.3, O87.1, I26.0 and I26.9 | 1094, 1093 |
| Coronary heart disease | I20, I21, I22, I23, I24, I25 | 1066, 1074, 1075 |
| Stroke | I60, I61, I63, I64 | 1081, 1086, 1491 |
| atrial fibrillation | I48 | 1471, 1483 |
| Chronic kidney disease | N03, N04, N05, N07, N11,  N12, N13, N14, N15, N18, N19 | 1192, 1427, 1519 |
| Valvular heart disease |  |  |
| Aortic valve stenosis | I35.0, I35.2 | 1490 |
| Mitral valve regurgitation | I34.0 | 1585 |
| Aortic valve regurgitation | I35.1 | 1587 |

**Note:** ICD, International Classification of Diseases

**Supplementary Table 2. Baseline characteristics of participants stratified by pancreas PDFF tertile.**

| Variables | Tertile 1 | Tertile 2 | Tertile 3 | *p* value |
| --- | --- | --- | --- | --- |
| n | 7346 | 7346 | 7346 |  |
| Age, years, mean (SD) | 52.76 (7.34) | 54.73 (7.37) | 56.79 (7.09) | <0.001 |
| Sex, n (%) |  |  |  | <0.001 |
| Female | 5256 (71.50) | 3562 (48.50) | 2536 (34.50) |  |
| Male | 2090 (28.50) | 3784 (51.50) | 4810 (65.50) |  |
| White, n (%) | 7065 (96.20) | 7134 (97.10) | 7197 (98.00) | <0.001 |
| University education degree, n (%) | 3862 (52.60) | 3359 (45.70) | 3050 (41.50) | <0.001 |
| Systolic blood pressure, mm/Hg, mean (SD) | 132.07 (18.49) | 137.57 (18.74) | 140.62 (18.28) | <0.001 |
| Diastolic blood pressure, mm/Hg, mean (SD) | 78.72 (10.26) | 81.85 (10.36) | 83.68 (10.13) | <0.001 |
| HbA1c, mmol/mol, Mean (SD) | 34.02 (4.20) | 34.94 (5.05) | 35.96 (5.89) | <0.001 |
| Smokers, n (%) | 2339 (31.8) | 2909 (39.6) | 3293 (44.8) | <0.001 |
| Drinkers, n (%) | 7137 (97.20) | 7164 (97.50) | 7180 (97.70) | 0.074 |
| Coronary heart disease, n (%) | 94 (1.30) | 179 (2.40) | 332 (4.50) | <0.001 |
| eGFR, mL/min/1.73m^2^, mean (SD) | 93.98 (11.81) | 91.89 (12.02) | 89.99 (12.28) | <0.001 |
| BMI，kg/m^2^, Mean (SD) | 24.36 (3.32) | 26.79 (3.83) | 28.54 (4.15) | <0.001 |
| C reactive protein, mg/L, mean (SD) | 1.43 (2.72) | 2.04 (3.32) | 2.56 (3.87) | <0.001 |
| Total Cholesterol, mmol/L, Mean (SD) | 5.66 (1.02) | 5.78 (1.09) | 5.74 (1.12) | <0.001 |
| Low-Density Lipoprotein Cholesterol, mmol/L, Mean (SD) | 3.47 (0.78) | 3.64 (0.84) | 3.65 (0.85) | <0.001 |
| Chronic kidney disease, n (%) | 19 (0.25) | 26 (0.35) | 33 (0.44) | 0.151 |
| Stroke, n (%) | 34 (0.46) | 35 (0.47) | 72 (0.98) | <0.001 |
| Hypertension, n (%) | 863 (11.70) | 1442 (19.60) | 2037 (27.70) | <0.001 |
| Valvular heart disease, n (%) | 57 (0.77) | 41 (0.55) | 56 (0.76) | 0.207 |
| Atrial fibrillation, n (%) | 22 (0.29) | 40 (0.54) | 65 (0.88) | <0.001 |
| Venous thromboembolism, n (%) | 62 (0.84) | 92 (1.25) | 101 (1.37) | 0.007 |
| Incident Heart Failure, n (%) | 37 (0.50) | 87 (1.18) | 158 (2.15) | <0.001 |

**Note:** Data are presented as number (percentage) or mean (standard deviation, SD).

Abbreviations: eGFR, estimated glomerular filtration rate; HbA1c, Hemoglobin A1c; BMI, body mass index, PDFF, proton density fat fraction.

**Supplementary Table 3. Baseline characteristics of participants stratified by liver PDFF tertile.**

| Variables | Tertile 1 | Tertile 2 | Tertile 3 | *p* value |
| --- | --- | --- | --- | --- |
| n | 7346 | 7346 | 7346 |  |
| Age, years, mean (SD) | 53.85 (7.54) | 55.44 (7.42) | 54.99 (7.30) | <0.001 |
| Sex, n (%) |  |  |  | <0.001 |
| Female | 5108 (69.50) | 3384 (46.10) | 2862 (39.00) |  |
| Male | 2238 (30.50) | 3962 (53.90) | 4484 (61.00) |  |
| White, n (%) | 7146 (97.3) | 7136 (97.1) | 7114 (96.8) | 0.275 |
| University education degree, n (%) | 3865 (52.60) | 3441 (46.80) | 2965 (40.40) | <0.001 |
| Systolic blood pressure, mm/Hg, mean (SD) | 131.96 (18.55) | 137.53 (18.70) | 140.78 (18.20) | <0.001 |
| Diastolic blood pressure, mm/Hg, mean (SD) | 78.29 (9.94) | 81.57 (10.28) | 84.39 (10.24) | <0.001 |
| HbA1c, mmol/mol, Mean (SD) | 34.12 (4.05) | 34.78 (4.72) | 36.01 (6.26) | <0.001 |
| Smokers, n (%) | 2450 (33.4) | 2925 (39.8) | 3166 (43.1) | <0.001 |
| Drinkers, n (%) | 7155 (97.4) | 7178 (97.7) | 7148 (97.3) | 0.256 |
| Coronary heart disease, n (%) | 139 (1.89) | 194 (2.64) | 272 (3.70) | <0.001 |
| eGFR, mL/min/1.73m^2^, mean (SD) | 93.01 (12.18) | 91.25 (12.10) | 91.60 (12.10) | <0.001 |
| BMI，kg/m^2^, Mean (SD) | 24.28 (3.28) | 26.54 (3.63) | 28.86 (4.15) | <0.001 |
| C reactive protein, mg/L, mean (SD) | 1.47 (2.93) | 1.90 (3.23) | 2.65 (3.78) | <0.001 |
| Total Cholesterol, mmol/L, Mean (SD) | 5.65 (1.03) | 5.76 (1.07) | 5.77 (1.12) | <0.001 |
| Low-Density Lipoprotein Cholesterol, mmol/L, Mean (SD) | 3.45 (0.78) | 3.62 (0.83) | 3.68 (0.85) | <0.001 |
| Chronic kidney disease, n (%) | 24 (0.32) | 25 (0.34) | 29 (0.39) | 0.763 |
| Stroke, n (%) | 34 (0.46) | 44 (0.59) | 63 (0.85) | 0.010 |
| Hypertension, n (%) | 845 (11.5) | 1431 (19.5) | 2066 (28.1) | <0.001 |
| Valvular heart disease, n (%) | 51 (0.69) | 57 (0.77) | 46 (0.62) | 0.552 |
| Atrial fibrillation, n (%) | 33 (0.44) | 59 (0.80) | 35 (0.47) | 0.007 |
| Venous thromboembolism, n (%) | 77 (1.04) | 76 (1.03) | 102 (1.38) | 0.076 |
| Incident Heart Failure, n (%) | 71 (0.96) | 97 (1.32) | 114 (1.55) | 0.002 |

**Note:** Data are presented as number (percentage) or mean (standard deviation, SD).

Abbreviations: eGFR, estimated glomerular filtration rate; HbA1c, Hemoglobin A1c; BMI, body mass index, PDFF, proton density fat fraction.

**Supplementary Table 4. Baseline characteristics of participants stratified by visceral fat volume tertile.**

| Variables | Tertile 1 | Tertile 2 | Tertile 3 | *p* value |
| --- | --- | --- | --- | --- |
| n | 7346 | 7346 | 7346 |  |
| Age, years, mean (SD) | 53.60 (7.51) | 55.04 (7.39) | 55.64 (7.31) | <0.001 |
| Sex, n (%) |  |  |  | <0.001 |
| Female | 5783 (78.70) | 4161 (56.60) | 1410 (19.20) |  |
| Male | 1563 (21.30) | 3185 (43.40) | 5936 (80.80) |  |
| White, n (%) | 7103 (96.7) | 7092 (96.5) | 7201 (98.0) | <0.001 |
| University education degree, n (%) | 3946 (53.70) | 3334 (45.40) | 2991 (40.70) | <0.001 |
| Systolic blood pressure, mm/Hg, mean (SD) | 131.25 (18.69) | 136.95 (18.27) | 142.06 (17.98) | <0.001 |
| Diastolic blood pressure, mm/Hg, mean (SD) | 77.66 (9.94) | 81.39 (10.01) | 85.19 (10.03) | <0.001 |
| HbA1c, mmol/mol, Mean (SD) | 34.03 (3.88) | 34.89 (4.75) | 35.99 (6.35) | <0.001 |
| Smokers, n (%) | 2395 (32.60) | 2754 (37.50) | 3392 (46.20) | <0.001 |
| Drinkers, n (%) | 7127 (97.00) | 7141 (97.20) | 7213 (98.20) | <0.001 |
| Coronary heart disease, n (%) | 82 (1.11) | 180 (2.45) | 343 (4.66) | <0.001 |
| eGFR, mL/min/1.73m^2^, mean (SD) | 93.52 (11.81) | 91.61 (12.11) | 90.73 (12.36) | <0.001 |
| BMI，kg/m^2^, Mean (SD) | 23.71 (2.89) | 26.62 (3.43) | 29.35 (3.94) | <0.001 |
| C reactive protein, mg/L, mean (SD) | 1.36 (2.82) | 2.06 (3.46) | 2.61 (3.65) | <0.001 |
| Total Cholesterol, mmol/L, Mean (SD) | 5.67 (1.02) | 5.81 (1.08) | 5.70 (1.13) | <0.001 |
| Low-Density Lipoprotein Cholesterol, mmol/L, Mean (SD) | 3.44 (0.77) | 3.67 (0.82) | 3.65 (0.86) | <0.001 |
| Chronic kidney disease, n (%) | 17 (0.23) | 21 (0.28) | 40 (0.54) | 0.003 |
| Stroke, n (%) | 28 (0.38) | 52 (0.70) | 61 (0.83) | 0.002 |
| Hypertension, n (%) | 708 (9.60) | 1321 (18.00) | 2313 (31.50) | <0.001 |
| Valvular heart disease, n (%) | 53 (0.72) | 56 (0.76) | 45 (0.61) | 0.530 |
| Atrial fibrillation, n (%) | 17 (0.23) | 57 (0.77) | 53 (0.72) | <0.001 |
| Venous thromboembolism, n (%) | 70 (0.95) | 88 (1.19) | 97 (1.30) | 0.105 |
| Incident Heart Failure, n (%) | 33 (0.44) | 94 (1.27) | 155 (2.10) | <0.001 |

**Note:** Data are presented as number (percentage) or mean (standard deviation, SD).

Abbreviations: eGFR, estimated glomerular filtration rate; HbA1c, Hemoglobin A1c; BMI, body mass index, PDFF, proton density fat fraction.

**Supplementary Table 5. Sensitivity analysis of the associations between visceral fat and heart failure.**

|  | HR (95% CI) | *p* value | Tertiles 1  HR (95% CI) | Tertiles 2  HR (95% CI) | Tertiles 3  HR (95% CI) | *p* trend |
| --- | --- | --- | --- | --- | --- | --- |
| Sensitivity 1 |  |  |  |  |  |  |
| Liver PDFF | 0.99 (0.97-1.02) | 0.638 | Reference | 0.86 (0.64-1.16) | 0.86 (0.64-1.16) | 0.375 |
| Pancreas PDFF | 1.02 (1.01-1.03) | <0.001 | Reference | 1.39 (0.97-2) | 1.66 (1.17-2.37) | 0.004 |
| Visceral Fat Volume | 1.09 (1.04-1.15) | <0.001 | Reference | 1.68 (1.16-2.44) | 1.76 (1.19-2.59) | 0.011 |
| Sensitivity 2 |  |  |  |  |  |  |
| Liver PDFF | 1.00 (0.97-1.02) | 0.815 | Reference | 0.83 (0.62-1.11) | 0.87 (0.65-1.16) | 0.348 |
| Pancreas PDFF | 1.02 (1.01-1.03) | 0.002 | Reference | 1.34 (0.93-1.91) | 1.58 (1.12-2.24) | 0.009 |
| Visceral Fat Volume | 1.08 (1.03-1.14) | 0.002 | Reference | 1.67 (1.15-2.42) | 1.73 (1.17-2.55) | 0.006 |

**Note:** Sensitivity 1: Sensitivity analysis of the associations between visceral fat and heart failure after excluding participants with follow–up duration less than 2 Years. Sensitivity 2: Sensitivity analysis of the associations between visceral fat and heart failure after adjusted for age, sex, race, education, current smoking, current drinking, systolic blood pressure, diastolic blood pressure, HbA1c, C-reactive protein, estimated glomerular filtration rate, TC, LDL-C, coronary heart disease, chronic kidney disease, stroke, atrial fibrillation, valvular heart disease and venous thromboembolism. The continuous visceral fat was quantified by: 1-point increment in Liver PDFF, 1-point increment in Pancreas PDFF, 1-point increment in Visceral Fat Volume, HR, hazard ratio; CI, confidence interval; PDFF, Proton Density Fat Fraction.

**Supplementary Fig. 1. Association between tertiles of visceral fat and incident heart failure.**

**Note:** (A) Liver fat determined from magnetic resonance imaging -derived proton-density fat fraction, with tertile 1 defined as low liver fat. (B) Pancreas fat determined from magnetic resonance imaging -derived proton-density fat fraction, with tertile 1 defined as low pancreas fat. (C) Total visceral fat determined from magnetic resonance imaging -derived visceral fat volume, with tertile 1 defined as low total visceral fat. PDFF, Proton Density Fat Fraction.
